# Isolation of Polyclonal Single-Chain Fragment Variable (scFv) Antibodies Against Venomous Snakes of Iran and Evaluation of Their Capability in Neutralizing the Venom

**DOI:** 10.22037/ijpr.2019.14400.12358

**Published:** 2020

**Authors:** Maryam Kadkhodazadeh, Masoumeh Rajabibazl, Mohammad Motedayen, Solmaz Shahidi, Ziba Veisi Malekshahi, Azam Rahimpour, Maral Yarahmadi

**Affiliations:** a *Department of Clinical Biochemistry, Faculty of Medicine, Shahid Beheshti University of Medical Sciences, Tehran, Iran. *; b *Department of Tissue Engineering and Applied Cell Sciences, School of Advanced Technologies in Medicine, Shahid Beheshti University of Medical Sciences, Tehran, Iran. *; c *Department of Serotherapy, Razi Vaccine and Serum Research Institute, Agricultural Research, Education and Extension Organization (AREEO), Karaj, Iran. *; d *Department of Medical Biotechnology, School of Advanced Technologies in Medicine, Tehran University, Tehran, Iran.*

**Keywords:** ScFv, Neutralization, Venom, Snake, Phage display

## Abstract

Several species of dangerous snakes are found in Iran and, according to the Emergency Response Center of Iran from 2002 to 2011, 53,787 Iranians have suffered from snakebite. Although the mortalities caused by snakebite are very low, snakebite-related amputations are still a major concern. Currently, anti-venom polyclonal antibodies derived from animals, such as horses are used to treat snakebites; however, in some cases they can cause anaphylactic shock and serum sickness. In line with this premise, generation of recombinant anti-venom antibodies can be considered as an alternative strategy. Single-chain fragment variable (scFv) antibodies offer several advantages compared to the whole antibodies, including ease of production, high affinity and specificity. In the present study, scFv antibodies were selected against the venom of the most poisonous snakes in Iran using phage display technology. Phage particles harboring anti-venom specific scFv were separated and scFv antibodies were produced in bacteria. *In-vitro* assay showed that polyclonal scFvs specifically bind to the venom. Furthermore, *in-vivo* experiment in mice BALB/c indicated effective toxin neutralization using 20 µg of polyclonal scFv. Our study indicates the neutralizing capacity of anti-venom polyclonal scFvs, although further neutralization assays are needed to confirm their effectiveness.

## Introduction

Envenoming following snakebite is a critical public health problem in tropical regions particularly Africa, Asia and Middle East (1). It has been estimated that more than 5 million people in the world are bitten by a snake, annually (2). 4500-6500 Iranian people are suffering from snakebite every year and the mortality rate in the victims has been reported to be 3-9 people (3).

Snake venom is composed of neurotoxic, hemotoxic, cytotoxic, and cardiotoxic components (4). Neurotoxin compounds attack the nervous system and cause paralysis in the envenomed victims (5). Cytotoxic and anticoagulant compounds lead to swelling, necrosis, and bleeding while cardiotoxic compounds cause heart electrophysiology disorder or muscle damage (6, 7). In terms of toxicity, venomous snakes often belong to the Elapidae and Viperidae families (8).

In Iran, the most dangerous and poisonous snakes are *Pseudocerastes persicus*, *Vipera albicornuta *and lebetina species belonging to the Viperidae family, and the *Naja naja oxiana* which belongs to the Elapidae family (9). Snakes of Viperidae family usually cause coagulopathy while elapids often cause nervous disorders (8). Among the above mentioned snakes *Naja naja oxiana* is considered as one of the most dangerous snakes in the world (10). The rate of deaths from snakebites by this snake has been reported 80 percent of untreated victims (4, 11). Therefore, development of effective strategies for neutralization of venom in victims is essential due to the high incidence of snakebite and abundance of venomous snakes in Iran. 

Snake venom contains various proteins and phospholipases that are poisonous for human. currently, polyclonal antibodies obtained from the immunized animals, including horse and sheep are used to treat snakebite victims (12). However, only 20% of the horse or sheep antibodies are specific to the poisonous components of which less than 5% can neutralize the toxin. As the result, a large amount of anti-venom antibody should be administered to the affected individuals to be effective (13). On the other hand, production of high amounts of polyclonal anti-venom antibodies requires continuous measurement of immunization and bloodletting from the host animals, which is inconsistent with the animal rights. 

In recent years, due to limitations in anti-serum therapy, utilization of recombinant antibodies such as scFv and nanobody has been considered as an alternative approach (14). Antibody fragments are routinely generated using the phage display technology (15). In this technology, antibody fragments which are encoded by the phage genome are expressed on the surface of the phage, therefore a direct link exists between the genotype and phenotype which further facilitates isolation and propagation of recombinant phages containing high affinity antibodies (16).

One of the first reports of generation of anti-venom scFvs was presented by Cardoso et al. in 2000 where a human scFv specific for crotoxin, a phospholipase A2 neurotoxin, was isolated from a naïve phage library. This scFv showed *in-vivo* neutralizing activity against the venom toxin (17). In another study a human scFv against phospholipase A2 (PLA2) in Bothrops venom was developed by Roncolato *et al*. They showed that selected scFv can partially inhibit PLA2 activity *in-vitro*, while myotoxicity reduction and increased survival of animals after venom reception was also indicated (18).

scFvs lack the constant Fc region found in whole antibodies, therefore, immunological reactions caused by the classic antibody do not exist. Also, the molecular weight of scFv fragments is reduced to only 30 KDa (19). In addition, scFvs offer other advantages including high binding affinity and specificity and tissue permeability (20). Therefore, in the current study we tried to isolate and produce polyclonal human scFvs to neutralize the combination of *Naja naja oxiana*, *Cerastes cerastes gasperettii*, *Echis carinatus sochureki*, *Vipera lebetina obtusa*, *Agkistrodon intermedius caucasicus,* and *Vipera xanthina *venoms. 

## Experimental

*Preparation of scFv phagemid library *

 scFv phagemid library was achieved from Tomlinson I (Life Sciences, Cambridge, UK) and transformed into E. coli TG1 bacteria. E. coli TG1 was cultured in a 30 mL SB medium (Super broth: 5 gr Yeast extract, 10 gr Tryptone and 10 gr NaCl for 1 liter) containing 100 µg/mL ampicillin. For emancipation of phage particles expressing scFv antibodies, when the OD_600_ reached 0.6, 10^12 ^pfu M13K07 helper phage (Amersham Pharmacia Biotech, USA) was added and the culture was incubated in 37 °C for 30 min followed by another 30 min at 37 °C/250 rpm. Then, kanamycin was added at the final concentration of 70 µg/mL and the culture was incubated for 16 h at 37 °C/250 rpm. For separating the recombinant phage particles, the culture was centrifuged at 5000 rpm for 20 min and the supernatant was collected. Then 20% (v/v) PEG solution (polyethylene glycol 6000 in 2.5 M NaCl) was added to the supernatant and incubated for 4 h on ice. In order to eliminate the bacteria cells from supernatant, the supernatant was incubated in water bath at 60 °C for 20 min and bacterial debris were removed by centrifugation at 5000 rpm for 5 min. The solution was centrifuged at 4 °C /12,000 rpm for 20 min and pellet containing phage particles was resuspended in TBS (50 mM Tris–Cl, 150 mM NaCl, pH 7.5) solution containing 1% BSA. Finally, the supernatant was stored at 4 °C until the next use. 

*Bio-panning and polyclonal phage-ELISA*

 For the first round, 10 μg of the venom and 10 μg BSA were coated in microplate in duplicate and incubated for 16 h at 4 °C. The wells were washed three times with TBST 0.05% (TBS + 0.05% Tween-20) solution and blocked with 200 µL TBS buffer containing 3% BSA, then incubated for 2 h at 37 °C. After washing, 100 µL of recombinant phage library was added to wells containing BSA and incubated for 1 h at a temperature of 37 °C. Then the recombinant phage library solution from wells containing BSA was collected and added to wells containing the toxin and incubated for 1 h at 37 °C. In the next step, the wells were washed several times with TBST and the conjugated phages were eluted with 100 µL of Glycine-HCl (pH = 2.2). After 10 min, the released phages were neutralized with 40 µL of Tris buffer (pH = 9.2) and added to 30 mL of TG1 bacteria culture with OD_600 _of 0.6. The phages were propagated as described under “Preparation of scFv phagemid library”. The separated phages were used for the next round of panning. Five rounds of bio-panning were carried out with the toxin concentrations of 10, 5, 2.5, and 1.25 µg. In order to perform polyclonal phage ELISA, each well of the microtitre plate was coated with 10 µg of venom and BSA (as a non-specific antigen) and incubated at 4 °C for 16 h. The wells were washed and blocked as described before. Separated phages from each round of panning were added to corresponding wells and incubated at 37 °C for 2 h. After washing using TBST 0.05%, diluted anti-M13 monoclonal antibody conjugated to HRP (Amersham, Germany) was added to the wells and incubated at 37 °C for 2 h. the wells were washed three times using TBST 0.05% and TMB (3, 3′, 5, 5′-Tetramethylbenzidin) substrate (Sigma, USA) was added to the wells. After 20 min the reaction stopped with 3 N H_2_SO_4_ solution. Finally, the optical density was read at 450 nm. 

*Expression and purification of selected polyclonal scFv *

 Phagemids were extracted by plasmid extraction kit (Bioneer, South Korea) and transformed into HB2151 bacteria. The bacteria were grown in LB (Luria Bertani) medium until the culture reached an OD_600 _of 0.8. Expression of the polyclonal scFv was optimized with 1mM IPTG at 37 °C for 5 h. the bacterial cells were collected by centrifugation at 5000 rpm for 20 min and after adding 6 M urea solution to pellet, periplasmic proteins were extracted by sonication. Purification of scFv antibodies was performed using Ni-NTA chromatography (Qiagen, Germany). Results of the expression and purification were surveyed on 12% SDS PAGE (Figure2). 

*Rat immunization and serum semi purification*

 3-month-old rats were used for immunization. At first 200 µL of 250 µg/mL toxin solution was combined with 200 µL of Freund’s complete adjuvant and was injected intraperitoneally (IP). Subsequent immunizations were performed by a mixture of 200 µL of venom (at the concentration of 250 µg/mL) and 200 µL of incomplete Freund’s adjuvant, injected on days 10, 22, and 36 after first injection, respectively. To evaluate rat immunization, seven days after the fourth injection, 200 µL of bloods were collected and the antibody’s titer was determined using ELISA. Then the bloods from the control and immunized rats were collected and fractionated with ammonium sulfate. The amount of ammonium sulfate was calculated by the ammonium sulfate calculator software (http://www.encorbio.com). The results of semi-purification and immunization were analyzed on 7% SDS PAGE and ELISA, respectively. 96-Wells microtiter plate was coated with either 1.25 µg of venom or BSA in 100 µL PBS and incubated at 4 °C for 16 h. Then the wells were washed and blocked as previously was described. Dilutions of 1/500, 1/2000, and 1/5000 of immune sera and 1/300 of non-immune sera were prepared with PBS and added to the wells and were incubated at 37 °C for 1 h. The wells were washed with TBST 0.05% and then incubated with HRP conjugated anti-rat antibody (1/20000 in PBS) (Abcam, UK) for 1h at 37 °C. Following the final washing step, TMB was added to each well and after 20 min, the reaction was stopped with 3N H_2_SO_4 _solution. The optical density of each reaction was measured at 450 nm. 

*Evaluation of antibody binding*

Venom in concentrations of 0.25, 1, and 10 µg per 100 µL PBS was coated in a 96-well microtiter plate in duplicate and incubated at 4 °C for 16 h. The following steps were carried out as described for polyclonal phage-ELISA except that purified scFv in concentration of 5 µg per 100 µL of PBS was used instead of phage, and anti-His antibodies conjugated with HRP (Abcam, UK) were applied instead of anti-M13 antibodies conjugated with HRP. 

*Evaluation Specificity of polyclonal scFv by Sandwich ELISA*

The 96-Wells microtiter plate was coated in duplicate with the scFv as the capture antibody at a concentration of 5 μg/mL in PBS and was incubated at 4 °C for 16 h. Then wells were washed and blocked as previously described for polyclonal phage-ELISA (21). 100 µL of venom and BSA in concentration of 5 μg/mL in PBS were added to the corresponding wells. The wells were washed and subsequently incubated with 1/1000 and 1/300 dilutions of immune and non-immune sera, respectively, and were incubated at 37 °C for 1 h. The rest of the steps were carried out as previously described under rat immunization and semi-purification serum.

*Evaluation of LD*_50_

The toxin was provided by Razi Institute. Dilutions for injection were prepared from the stock solution with 1.2 µg/µL concentration. BALB/c mice with 15-gr weight were divided to three groups and injected with 200 µL of toxin dilutions intraperitoneally in duplicates. The lethality was monitored 72 h post-injection and the toxin concentration in which 50% of the mice were survived was selected as LD_50_. 

*In-vivo Challenge of the Mice*

 First, lethal dose of venom (LD_50_) was determined. The LD_50_ of the venom was indicated 8.5 µg in BALB/c mice weighing 15 gr. To appraise the neutralization capability of the purified scFv, different concentrations of scFv and venom were injected intraperitoneally. The mice were divided into six groups. Venoms in concentrations >1 LD_50_ and 2 LD_50_ were mixed with different concentrations of scFv and after incubation at 37 °C for 1 h, were injected into the mice. The survival of the mice was monitored after 72 h. 

## Results

*Panning*

**Figure1:**
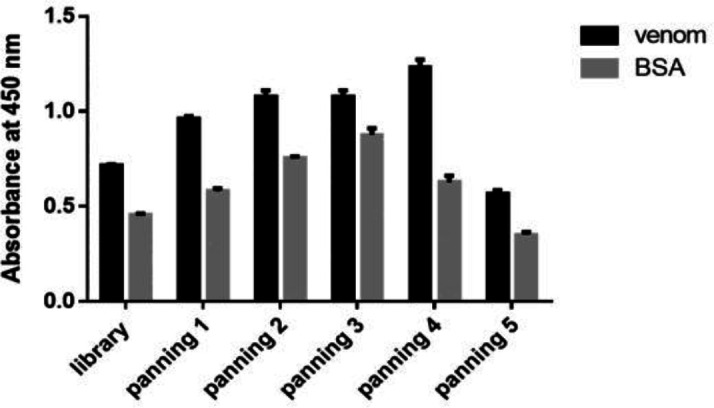
Results of polyclonal-phage ELISA. Phages obtained after each round of panning were tested against venom by ELISA. Fourth Round showed the highest absorbance and was used for expression of monovalent scFv antibodies in soluble form

The recombinant scFv phagemid library was used for five round of panning against venom. The result of polyclonal phage-ELISA showed that fourth round of panning had antibody fragments with the highest affinity against toxin (Figure 1) and was used for expression of monovalent scFv antibodies in soluble form.

*Expression and purification of scFv antibodies *

**Figure 2:**
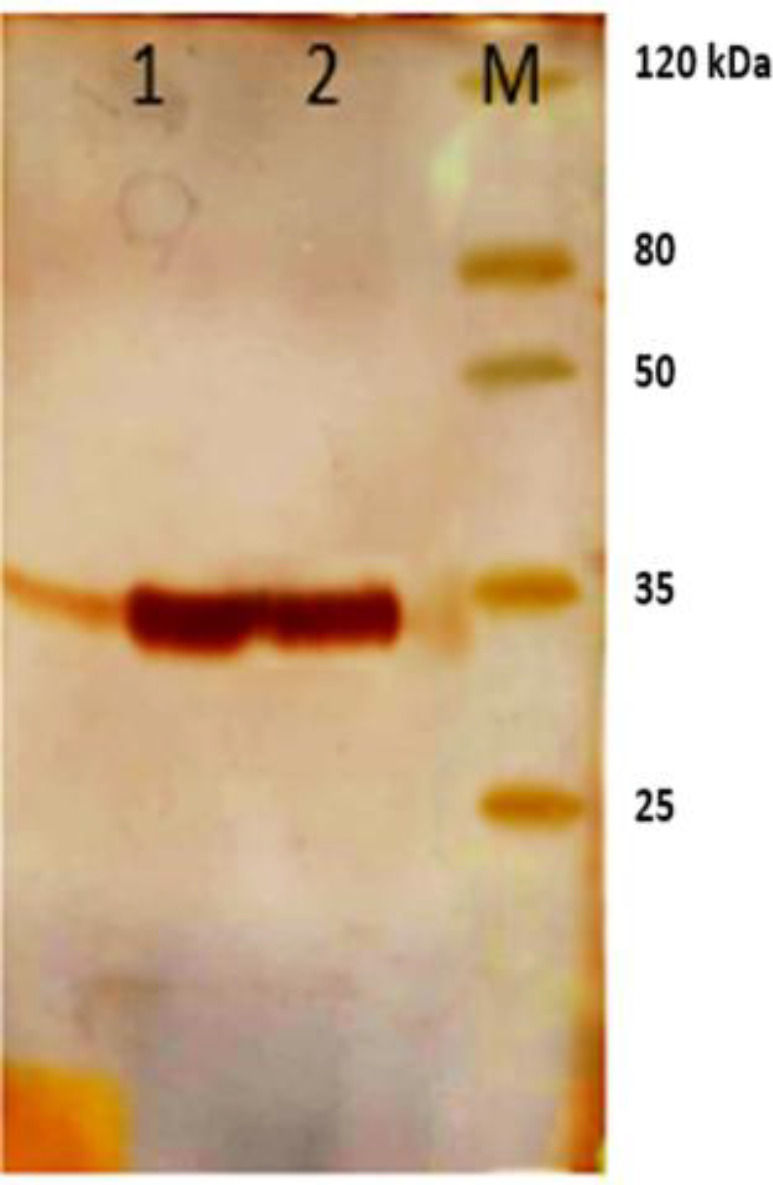
Analysis of polyclonal scFv purification on SDS PAGE. Line M: standard protein marker. Line 1, 2: column outcome after elution with native buffer B (Qiagen) containing 200 mM imidazole

 scFv antibodies obtained from fourth round of panning were expressed in a non-suppressor E. coli HB2151 strain. Following cell disruption and precipitation of the cell debris, the scFv fragments were purified using Ni-NTA affinity chromatography. A single band around 35 KDa corresponding to the scFv was detected on 12% SDS PAGE (Figure 2). 

*Rat immunization and serum semi purification*

**Figure 3:**
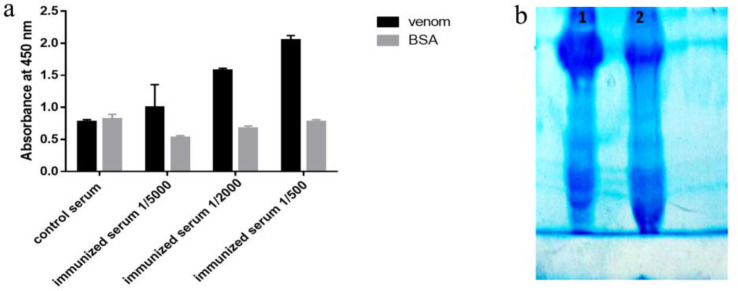
(A) Assessment of rat immunization after four stage venom injection by ELISA. The results show that in different dilutions of immunized serum, the absorption rate of toxin versus non-specific antigen (BSA) was significantly higher. (B) Result of semi-purification of polyclonal antibody surveyed on 7% SDS PAGE. Line 1: immunized serum Line 2: control serum

 To evaluate the rat immunization, 7 days after the fourth injection, antibody titer and its specificities to venom was determined using ELISA. The results of this analysis showed that the serum from immunized mice had higher absorbance and higher specificity than the toxin compared to the control mice (Figure 3a). Control and immunized rat bloods were collected and immunoglobulin’s precipitation was performed using ammonium sulfate. Semi-purified products were analyzed on 7% SDS PAGE (Figure 3b).

*Evaluation of antibody binding*

**Figure 4:**
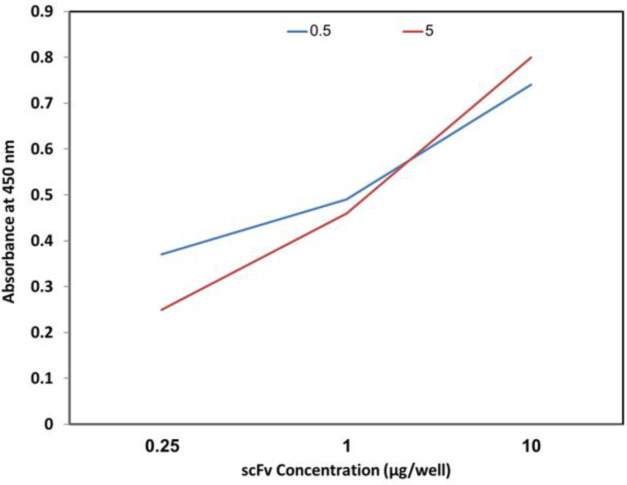
Evaluation of scFv bonding at 5 µg concentration to the venom at 0.25, 1 and 10 µg concentrations

Antibody binding to different concentrations of the venom was evaluated using different concentrations of the toxin. As it is shown in Figure 4, increasing the toxin concentration leads to the enhancement of the optical density. 

*scFv antibodies binding and specificity characterization*

**Figure 5:**
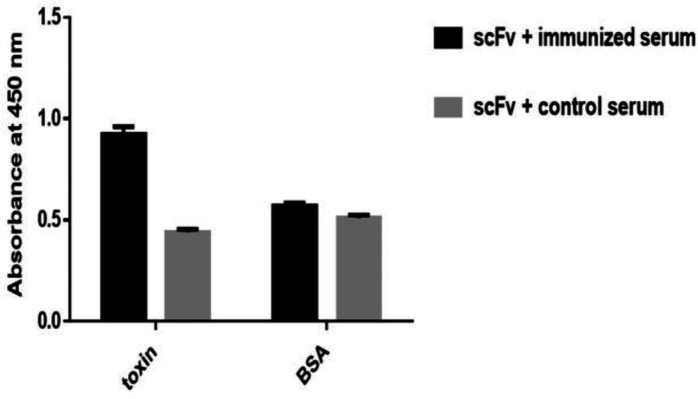
Specificity of scFv antibody. Venom and BSA was captured between polyclonal scFv and rat polyclonal antibodies

To evaluate the specificity of purified pooled scFv antibodies a sandwich ELISA was carried out using rat polyclonal antibody and scFv antibodies. scFv antibodies showed specific binding to the venom while no significant cross-reactivity with unrelated immobilized antigens (BSA) was detected (Figure 5). 

*Calculation of LD*_50_

LD_50_ was calculated for toxin at 1.25-10 µg/100 µL concentrations. The results of LD_50_ determination is indicated in Table 1. According to this test, toxin concentration of 8.75 µg per 100 µL was selected as LD_50_. 

*Neutralization assays*

One of the main objectives of this study was isolation of the human antibody fragments using phage display technology that could provide protection against the toxic effects of venom *in-vivo*. For this reason, the neutralization capacity of polyclonal scFv was tested in BALB/c mice. Venom was mixed with determined scFv concentration, incubated at 37 °C for 1 h and was injected through intraperitoneal injection. The results of the *in-vivo* experiments are shown in Table 2 According to these results, group 1 which was injected with 1 LD_50_ showed 80% survival rate, group 2 which was injected with 1.42 LD_50_ showed 75% survival rate. However, when 2 LD_50_ was used, only scFv concentration at 20 µg (group 5) could neutralize the toxin, which resulted in survival rate of 57%. 

## Discussion

Snakebite is a serious health concern in many tropical countries, especially in South Asia (22). Snakebite is a prevalent problem in Iran, particularly in rural areas. During 2002-2011, abundance of snakebites was reported 4.5-9.1 per 100,000 population (9). The most medically important snakes in Iran are *Naja naja oxiana*, *Cerastes cerastes gasperettii*, *Echis carinatus sochureki*, *Vipera lebetina obtusa*, *Agkistrodon intermedius caucasicus,* and *Vipera xanthina* (23). 

Elapids possess a potent neurotoxic venom which can cause muscle paralysis and death (24). Cardiotoxic components can also be found in Elapids venom. On the other hand, Vipers venom often contains a notable anticoagulant and cytotoxic trait which cause swelling and necrosis (25, 8). 

Currently, the available method for treatment of snakebite is based on the production of antibodies in horses immunized with the whole venom. The production of these antibodies is difficult and time-consuming and can lead to severe immunological reactions following patient’s response to the animal sera and disorders in the immune system that may lead to death. In line with this premise, development of recombinant antibodies and their fragments can be considered as an alternative strategy to neutralize snake venom. This strategy, can abstain long-term animal immunization protocols and reduces the risk for inducing immune responses in human (26). 

 In the present study, a combinatorial scFv library (Tomlinson I) was screened against the venom of six snake species. While previous studies have focused on generation of the recombinant antibodies against a toxic compound of the venoms (27), we sought to focus on the pool from the most poisonous compounds of snakes’ venom, listed before. Since the antigen used in this study was mixed venoms of six snake species, we decided to isolate polyclonal scFv instead of monoclonal scFv and examine finally its neutralization potential *in-vivo*. 

 To our knowledge, this is the first report on the development of polyclonal scFv binders against the snakes’ venom. For *in-vitro* selection of anti-venom, five rounds of panning were carried out against venom with increased stringency of the washing buffer and decreased amount of the venom. 

It was demonstrated that scFv-phage particles from the fourth stage of bio-panning had the highest affinity toward venom when compared to the non-specific antigen. Consequently, scFv-phage particles were derived from the fourth stage of bio-panning were used for the production of scFv in soluble phase (28). 

Sandwich ELISA indicated that our polyclonal scFv specifically recognizes venom; however, reasonably its specificity was less than monoclonal scFv fragments isolated against other antigens (20). Our results showed that polyclonal scFv at 20 µg concentration effectively neutralized the toxin upon intraperitoneal injection in challenged mice. As it was mentioned before, in this study, due to the use of an antigenic mixture and the subsequent production of polyclonal scFv, the results of specificity tests and the challenge of neutralization were variable. Subsequently, it is suggested that a mixture of monoclonal scFv be prepared and its ability to neutralize the mixed venom be evaluated. 

In conclusion, considering the advantages of scFv including the small size, the feasibility of their production in a variety of hosts including prokaryotic and eukaryotic cells, and reduced immunogenicity, they can be potent binders for venom neutralization. In addition, these antibody fragments have similar affinities as whole antibodies and they can be arranged into diabody and tribody formats. Therefore, scFvs can be suitable candidates for the development of therapeutic anti-venom antibodies (29, 19).

**Table 1:** Determination of the toxin LD50

**Toxin concentration** **µg/100 µL**	**Number of Live Mice**	**Number of dead Mice**
1.25	3	0
2.5	3	0
3.75	3	0
5	3	0
6.25	3	0
7.5	3	0
8.75	1	2
10	0	3

**Table 2:** *In-vivo* neutralization of venom with purified scFv fragments. LD50 of venom was indicated as 8.5 µg in BALB/c mice with 15 gr weight. Venom was mixed with determined scFv concentration and incubated at 37 °C at 1 h and injected intraperitoneally. The amount of scFv fragments needed for neutralization of the 1.5-fold venom was 20 µg for 15 gr mouse

**Group**	**Amount of scFv in 200 µL**	**Amount of venom in 200 µL**	**Survival ratio** **(alive/total)**	**Control** **(dead/total)**
1	5 µg	8.75 µg	4/5	2/2
2	20 µg	12.5 µg	3/4	2/2
3	20 µg	15 µg	2/2	1/1
4	15 µg	17 µg	0/2	1/1
5	20 µg	17 µg	4/7	2/2
6	25 µg	17 µg	1/2	1/1
